# Takotsubo Cardiomyopathy: Understanding the Pathophysiology of Selective Left Ventricular Involvement

**DOI:** 10.7759/cureus.5972

**Published:** 2019-10-23

**Authors:** Deepak Kumar Pasupula, Venkata Suresh Patthipati, Awais Javed, Sudeep K Siddappa Malleshappa

**Affiliations:** 1 Internal Medicine, University of Pittsburgh Medical Center, Pittsburgh, USA; 2 Internal Medicine, Trumbull Regional Medical Center, Warren, USA; 3 Internal Medicine, Baystate Medical Center, Springfield, USA

**Keywords:** takotsubo cardiomyopathy, pathophysiology, coronary microcirculation, adrenergic receptors, cholinergic receptors

## Abstract

Takotsubo cardiomyopathy (TCM) has gained global recognition as a unique cardiovascular disease that mimics acute myocardial infarction. Since its initial description, more than three decades ago, we have significantly advanced our understanding of diagnosing, treating, and prognosticating this reversible cardiovascular phenomenon. However, the pathophysiological explanation behind its selective involvement of the left ventricle (LV), predominantly the LV apex in poorly understood. In this brief review on differential distribution of the adrenergic nerve (AN) and cholinergic nerve (CN) in the normal human heart, we try to extrapolate an idea of poor CN distribution in the LV apex as an associated factor augmenting microcirculatory dysfunction due to an unopposed AN activity from the catecholamine surge, as a plausible explanation for this characteristic phenomenon.

## Introduction and background

Since 1990, after describing the term tako-tsubo-like left ventricular dysfunction, several concepts have been put forth to individually explain the unique contractile dysfunction seen in Takotsubo cardiomyopathy (TCM) [1-3]. In particular, the microcirculatory dysfunction theory is a widely accepted explanation in the genesis of TCM. Rivero et al., in a small group (n=14) of women, angiographically assessed the index microcirculatory resistance (IMR) as a marker of microvascular dysfunction and demonstrated an inverse correlation between extent of microvascular dysfunction and the time of symptom onset [4], adding to the existing evidence towards a significant association between the existence of microcirculation dysfunction and TCM pathophysiology [3,5-8].

Additionally, there is increasing evidence of microcirculatory dysfunction occurring predominantly during the acute phase of TCM [9-12]; as demonstrated in a small study group, administering intravenous adenosine among TCM patients during acute phase has shown a significant immediate improvement in myocardial perfusion and left ventricular ejection fraction [6], suggesting an intense microvascular constriction playing an important role in the pathophysiology of acute TCM. Microcirculation dysfunction is often demonstrated in the presence of normal macrovascular coronary blood flow, as assessed using coronary angiography [13-14], in-spite of a 12-lead electrocardiogram demonstrating ST-segment elevation [15]. Therefore, providing evidence towards altered blood flow at a certain level, likely in microcirculation.

Microcirculation dysfunction theory in addition to a few notable pathophysiological mechanisms like; multi-vessel epicardial spasm [16-17], transient ischemia due to coronary plaque rupture [13], and cardiotoxicity from catecholamine surge [18] would explain the pathophysiology of TCM, but fail to provide evidence towards selective involvement of LV apex. Therefore, in this brief scientific review, we generate a hypothesis of differential distribution of the adrenergic nerve (AN) and cholinergic nerve (CN) to not only understand the pathophysiology of TCM but also to learn its selective involvement of LV apex.

## Review

Kawano et al. examined six autopsied hearts of people who died of non-cardiac cause. Using differential staining for tyrosine hydroxylase and acetylcholinesterase, the authors demonstrated a disparity in the regional distribution of the adrenergic (sympathetic) and cholinergic (parasympathetic) nerves, respectively [19]. Although this study involves only six male hearts, the autopsied hearts were free from cardiovascular disease as frequently noticed in TCM and among the homogenous age group (50 - 68 years) during which TCM is most prevalent [3]. Both AN and CN were found to traverse from epicardium to myocardium accompanying coronary vasculature to reach the myocytes. Within myocardium, the sub-epicardial layer had more AN, while CN dominated the sub-endocardial layer. Among ventricles, the anterior wall had more AN when compared to the posterior wall.

**Figure 1:**
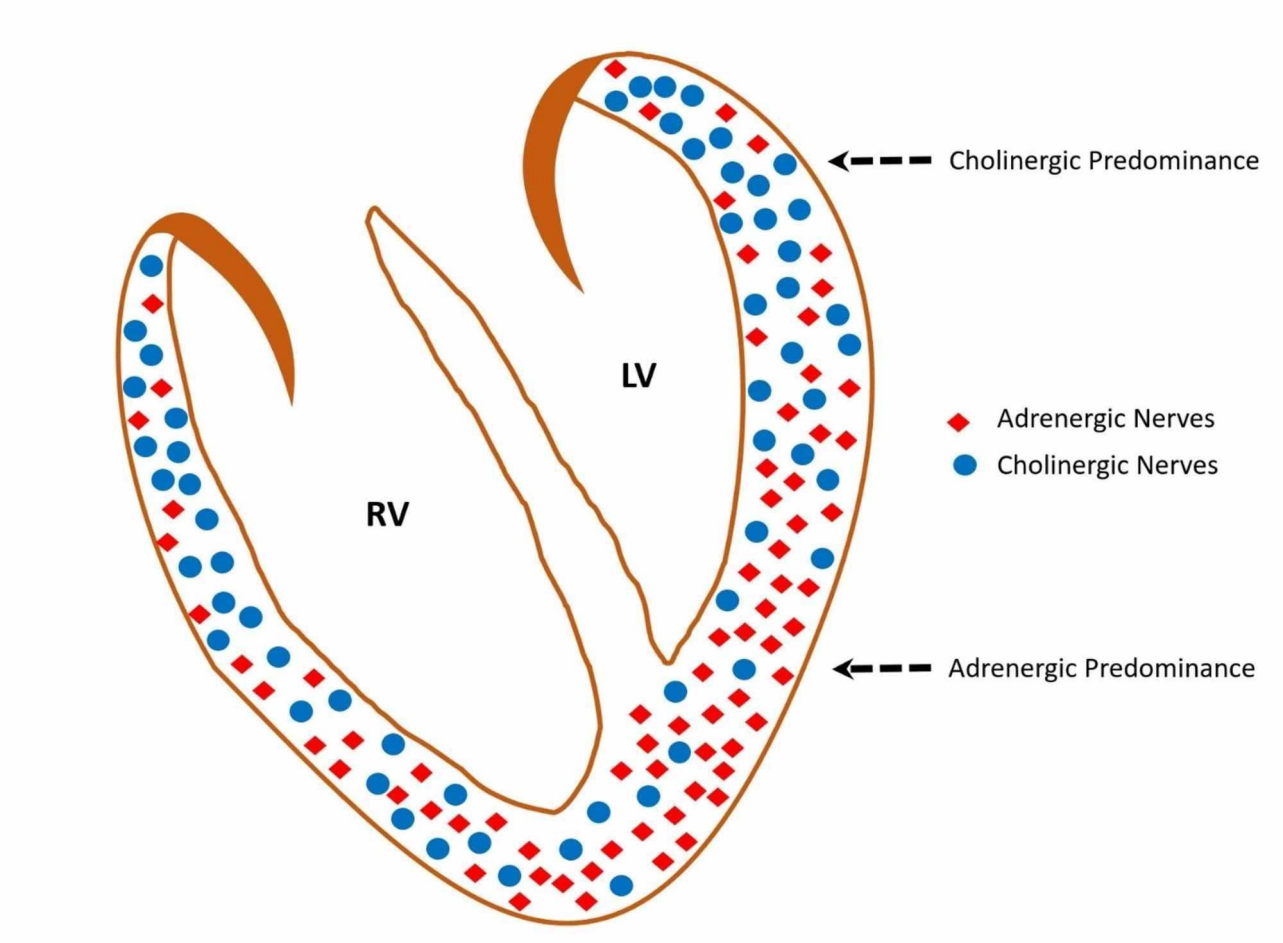
Schematic recreation of the adrenergic and cholinergic nerve distribution in normal ventricles A higher ratio of adrenergic nerve: cholinergic nerve is noted in the left ventricle apex which is due to the low density of cholinergic nerves. RV: right ventricle, LV:  left ventricle.

Atria had the highest concentration of CN in the heart, while ventricles were dominated by AN. Among ventricles (right versus left), there was no significant variation in the distribution of AN. Therefore, heterogeneous CN distribution between the ventricles significantly affects its regional microvascular blood flow by way of its vasodilatory mechanism. The left ventricle (LV) base and apex had a lower concentration of CN when compared to the concordant site in the right ventricle (RV), and in particular, LV apex had the lowest concentration of CN among all sub-regions of the heart (Figure 1). Myocardial microvascular blood flow is tightly regulated by maintaining an equilibrium between the vasoconstricting and vasodilatory effect of the AN and CN, respectively, via a G-protein signaling pathway [20].

Among TCM patients, an antecedent physical or emotionally stressful event causes a sudden surge in catecholamine level, which augments the AN activity causing intense coronary vasospasm [21]. This vasoconstriction is usually attenuated by CN mediated vasodilation. However, in regions of the heart, like the LV apex where there is less density of CN, vasoconstriction caused by an exponential catecholamine surge remains unopposed, causing myocardial ischemia. In females, with advancing age, there is a loss of protective vasodilatory effect from estrogen and there is a shift in cardiac autonomic function towards adrenergic dominance [22-23]. Therefore, females are at higher risk for multi-territorial myocardial ischemia leading to clinical manifestations similar to ST-elevation myocardial infarction, such as chest pain, troponin elevation, electrocardiographic and positron emission tomographic changes [24-26]. Since major coronary vessels traverse in the epicardium, they are minimally affected by this disequilibrium and therefore have normal coronary angiography and intact coronary vessel wall on intravascular ultrasound [13].

Lyon et al. proposed that during the catecholamine surge, norepinephrine causes a stimulating effect on the myocardial β1-adrenoreceptors (β1-ARs), resulting in increased contractility at the LV base. While epinephrine causes stimulation of β2-AR, leading to a switch from G(s) to G(i) protein pathway, causing a negative inotropic effect at the LV apex [27]. This theory can explain the apoptotic changes seen in the LV apical myocardium due to excessive stimulation of β1-ARs, although partly attenuated by the β2-AR-G(i) protein switch, the selective involvement of the LV apex is due to high concentration of β-AR receptors in the apex. Additionally, the stunned LV apex (due to β2-AR stimulation) is now subjected to the hypercontractile LV base (due to β1-AR stimulation), causing increased end-systolic LV pressure, leading to the ballooning effect of the LV apex.

Given that TCM is a constellation of mismatch between vasoconstriction and vasodilatory response, adenosine challenge during the acute phase of TCM causes a reversal of myocardial perfusion defect, increase in myocardial contractility and significant improvement in the ejection fraction when compared to a patient with ST-elevation MI who have a flow-limiting lesion [6]. Adenosine causes a Gs-protein mediated hyperpolarization of the vascular smooth muscle cells, leading to relaxation and vasodilation, which counteracts the intense vasoconstricting effect caused by catecholamine surge. In the event of chronotropic dominance and myocardial ischemia from norepinephrine surge, a switch in the cellular glucose metabolism from aerobic to the anaerobic occurs to meet the increasing energy demand.

Although this hypothesis complements previously published the theory of coronary microvascular dysfunction [28]. Our theory of disparity in AN: CN ratio at different regions of the heart helps us to better understand the physiology of microvascular dysfunction and its predilection towards LV apex with greater reasoning. Therefore, we can generate a hypothesis of heterogeneous distribution of the AN and CN in the heart as the basis for these collective manifestations seen in TCM. An epidemiological study, such as a case-control study in which myocardial biopsy of the TCM (cases) and healthy age and gender-matched individuals (control) can aid in testing this hypothesis. However, understanding the clinical complexity of a TCM patient, performing a myocardial biopsy in the acute phase will be a technical challenge and unethical. Therefore, animal models or assessing AN activity non-invasively using metaidobenzylguanidine (MIBG) scan and compare the regional distribution of AN in TCM and healthy subjects may be helpful, but it is important to understand that pathophysiology of TCM is linked to a low density of CN in the LV apex rather than understanding the distribution of AN. Probably animal studies to assess the ratio of AN and CN distribution among those who develop TCM as opposed to those who do not develop TCM after a stressor might help to test and establish this hypothesis.

## Conclusions

Takotsuko cardiomyopathy is a constellation of catecholamine surge with hypokinetic LV apex and functional hyperkinetic LV base, predominantly noted in older women with a preceding stressful event. The existence of poor CN distribution in the LV apex leading to a lack in the counteractive vasoconstriction in the cardiac microvascular caused by catecholamine surge via AN pathway can explain the phenomenon seen in TCM. Although this theory is based on the findings from the autopsied heart of healthy individuals, due to ethical and logistic reason this is difficult to prove with certainty in TCM patients. Further research is needed to non-invasively assess parasympathetic (CN distribution) activity in the heart among TCM patients.
